# Towards understanding leydigioma: do G protein-coupled estrogen receptor and peroxisome proliferator–activated receptor regulate lipid metabolism and steroidogenesis in Leydig cell tumors?

**DOI:** 10.1007/s00709-020-01488-y

**Published:** 2020-03-16

**Authors:** M. Kotula-Balak, E. Gorowska-Wojtowicz, A. Milon, P. Pawlicki, W. Tworzydlo, B. J. Płachno, I Krakowska, A. Hejmej, J. K. Wolski, B. Bilinska

**Affiliations:** 1 Department of Endocrinology, Institute of Zoology and Biomedical Research, Faculty of Biology, Jagiellonian University in Kraków, Poland, Gronostajowa 9, 30-387 Kraków, Poland; 2 University Centre of Veterinary Medicine, University of Agriculture in Kraków, Mickiewicza 24/28, 30-059 Kraków, Poland; 3 Department of Developmental Biology and Invertebrate Morphology, Institute of Zoology and Biomedical Research, Faculty of Biology, Jagiellonian University in Kraków, Poland, Gronostajowa 9, 30-387 Kraków, Poland; 4 Department of Plant Cytology and Embryology, Institute of Botany, Faculty of Biology, Jagiellonian University in Kraków, Poland, Gronostajowa 9, 30-387 Kraków, Poland; 5 nOvum Fertility Clinic, Bociania 13, 02-807 Warszawa, Poland

**Keywords:** G protein-coupled estrogen receptor, Peroxisome proliferator–activated receptor, Leydig cell tumor, Steroidogenesis-controlling molecules, Ultrastructure

## Abstract

Leydig cell tumors (LCT) are the most common type of testicular stromal tumor. Herein, we investigate the G protein-coupled estrogen receptor (GPER) and peroxisome proliferator–activated receptor (PPAR) implication in regulation of lipid homeostasis including the expression of steroidogenesis-controlling molecules in clinical specimens of LCTs and tumor Leydig cells (MA-10). We showed the general structure and morphology of LCTs by scanning electron and light microscopy. In LCTs, mRNA and protein analyses revealed increased expression of GPER and decreased expression of PPARα, β, and γ. Concomitantly, changes in expression pattern of the lutropin receptor (LHR), protein kinase A (PKA), perilipin (PLIN), hormone sensitive lipase (HSL), steroidogenic acute regulatory protein (StAR), translocator protein (TSPO), HMG-CoA synthase, and reductase (HMGCS, HMGCR) were observed. Using MA-10 cells treated with GPER and PPAR antagonists (alone and in combination), we demonstrated GPER-PPAR–mediated control of estradiol secretion via GPER-PPARα and cyclic guanosine monophosphate (cGMP) concentration via GPER-PPARγ. It is assumed that GPER and PPAR can crosstalk, and this can be altered in LCT, resulting in a perturbed lipid balance and steroidogenesis. In LCTs, the phosphatidylinositol-3-kinase (PI3K)-Akt-mTOR pathway was disturbed. Thus, PI3K-Akt-mTOR with cGMP can play a role in LCT outcome and biology including lipid metabolism.

## Introduction

Leydig cell tumor (LCT; leydigioma) is the most common non-germ cell gonadal tumor, accounting for 1–3% of all testicular tumors in adults and 4–9% in prepubertal children (Gheorghisan-Galateanu 2014). In recent years, a marked increase in the incidence of LCTs has been observed (14.7% of all testicular tumors removed). LCTs are usually benign tumors especially in infancy (Bertram et al. 1991), although local recurrence or metachronous tumors of the contralateral testis have also been described. Survival after diagnosis of primary LCTs ranged from 2 months to 17 years (Rich and Keating 2000). In prepubertal patients, even malignant LCTs are less likely to metastasize. Patients with LCTs usually have symptoms of testicular swelling or various endocrinological disruptions (Lai et al. 2015). Gynecomastia is the main clinical manifestation in adults, but it may also be clinically significant in affected children who undergo precocious puberty (Lejeune et al. 1998). Some cases of LCTs were linked with increased plasma estradiol concentrations (Huang et al. 2013). Moreover, infertility and azoospermia are not an unusual finding in these patients (Bozzini et al. 2017).

Radical orchiectomy is the current standard of care, but testis sparing surgery (TSS) (enucleation), in conjunction with intraoperative frozen section (FSE), has been recently attempted with promising results (Giacaglia et al. 2000). Prepubertal individuals and those with smaller tumors that lack evidence of malignancy are directly recommended for TSS.

The etiology of LCTs is unknown and appears heterogeneous. Furthermore, the molecular basis of LCTs is poorly understood. Some studies showed a possible role of genetic factors in LCT development. Interestingly, genetic mutations identified in children and adults were different and, in some cases, associated with other cancers (Carvajal-Carmona et al. 2006). In adults, it was observed that a somatic activating mutation in the guanine nucleotide-binding protein α gene may result in tumor development, leading to hyperactivity of sex steroid biosynthesis (Bertram et al. 1991). In addition, Carvajal-Carmona et al. (2006) reported an inherited fumarate hydratase mutation appears to cause tumor growth through activation of the hypoxia pathway. According to study of Lejeune et al. (1998), alterations in local stimuli, including Müllerian-inhibiting hormone, inhibin, growth factors, and temperature, may also create favorable conditions for initiation and development of LCTs.

Decreased Leydig cell function is common in men with reproductive disorders, including testicular dysgenesis syndrome (TDS). This syndrome is comprised of subfertility, cryptorchidism, hypospadias, and testicular cancer (Skakkebaek et al. 2001). Leydig cell impairment manifests as a decreased testosterone/lutropin (LH) ratio and the presence of Leydig cell micronodules in the testis (Holm et al. 2003). The number and size of micronodules increase with the severity of TDS (Lardone et al. 2013). Due to ultrastructural changes demonstrated in Leydig cells within micronodules (decreased smooth endoplasmic reticulum, irregularly indented nuclear membrane, decreased lipofuscin pigment granules, and Reinke crystals), failure of their maturation is suggested (Soerensen et al. 2016). In patients with germ cell tumors, Leydig cell hypertrophy and hyperplasia were linked to elevated levels of chorionic gonadotropin (Zimmerli and Hedinger 1991). Various chemicals induced Leydig cell hyperplasia via disruption of the hypothalamic-pituitary axis (Dirami et al. 1996). Alternatively, an excess of various hormones (e.g., estrogen, prolactin) produce elevated LH levels that excessively stimulate steroidogenic Leydig cell function (Greaves 2012). Overproliferation of Leydig cells may result in the synthesis of non-functional steroid hormones. Morphologically, no differences appear between spontaneous or chemically induced Leydig cell adenomas (Aoyama et al. 1998). However, there is no evidence as to whether increase of Leydig cell number may further develop into malignant Leydig cell tumor (Gould et al. 2007).

Mitogenicity associated with estrogen receptor–mediated cellular events is believed to be the mechanism by which estrogens contribute to tumorigenesis. Currently, implications of estrogen signaling via canonical estrogen receptors (ERs), G protein coupled membrane estrogen receptor (GPER), as well as estrogen-related receptors (ERRs) are recognized in animal and human Leydig cell tumorigenesis (Carpino et al. 2007; Tazi et al. 2008; Kotula-Balak et al. 2012, 2018a; Chimento et al. 2014). In human testis, Fietz et al. (2014, 2016) showed high levels of GPER mRNA expression in Leydig cells. However, GPER multiple fast signaling pathways are already described (Prossnitz and Barton 2011); they function is still not entirely known. Peroxisome proliferator–activated receptor (PPAR) belongs to the steroid family receptors and is also able to bind steroid hormones (Levin 2011). In amphibians, rodents, and humans, three forms of PPAR have been described to date: PPARα, PPARβ (also known as PPARδ), and PPARγ (Schmidt et al. 1992). PPARs target genes that encode enzymes involved in peroxisome and mitochondria function as well as those of fatty acids, apolipoproteins, and lipoprotein lipase. Little is known about PPARs in the male reproductive system. In rat testis, PPARs are mainly expressed in Leydig and Sertoli cells (Braissant et al. 1996). It was shown that some PPAR chemicals alter testosterone production (Harada et al. 2016), and their long-term administration results in Leydig cell tumor development in rats (Hess 2003).

Scarce data are available on the molecular and biochemical characteristics of LCTs. Maintaining an adequate hormonal balance within the testis is the basis for proper gonadal function, thus playing a pivotal role for blocking hormone-secreting Leydig cell tumor development (Seyfried and Shelton 2010).

It is worth noting that biosynthesis of sex steroids is multi-level, controlled process (Miller 2013). It requires the coordinated expression of number of genes, proteins of various function [receptors, e.g., lutropin receptor (LHR), enzymes, transporters, and regulators, e.g., translocator protein (TSPO), steroidogenic acute regulatory protein (StAR)), signaling molecules (e.g., protein kinase A (PKA)], and their regulators in response to LH stimulation. Moreover, for cellular steroidogenic function, global lipid homeostasis is crucial. Perilipin (PLIN), hormone sensitive lipase (HSL), and HMG-CoA synthase (HMGCS) as well as reductase (HMGCR) are members of a cell structural and enzymatic protein machinery controlling lipid homeostasis (Liu et al. 2012). Activation of lipid metabolism is an early event in tumorigenesis (Seyfried and Shelton 2010) however, the precise expression pattern of lipid balance-controlling molecules and their molecular mechanism remains poorly characterized.

This study aims to determine the potential link between GPER and PPAR and whether this interaction regulates lipid homeostasis in LCTs. To further investigate the relationship of Leydig cell tumorigenesis to these receptors while elucidating the effects of their interactions, mouse tumor Leydig cells (MA-10) were utilized.

## Materials and methods

### Tissue samples and ethical considerations

Residual tissues from testicular biopsy (microdissection testicular sperm extraction by Schlegel, 1998) were collected at the nOvum Fertility Clinic, Warsaw, Poland from patients (31–45 years old; *n* = 24) diagnosed due to azoospermia (micronodules LCTs were recognized during surgery). After evaluation by pathologists, patient written informed consent according to the approval regulations by the National Commission of Bioethics at the Jagiellonian University in Krakow, Poland, permit no. 1072.6120.218.2017 and in accordance with the Declaration of Helsinki specimens were used for the present study. Remaining tissue fragments were snap-frozen or fixed and paraffin-embedded and were stored and analyzed at the Department of Endocrinology, Institute of Zoology and Biomedical Research, Jagiellonian University in Krakow, Poland.

### Cell culture and treatments

The mouse Leydig cell line MA-10 was a generous gift from Dr. Mario Ascoli (University of Iowa, Iowa City, USA) and was maintained under standard technique (Ascoli 1981). The cells were grown in Waymouth’s media (Gibco, Grand Island, NY) supplemented with 12% horse serum and 50 mg/l of gentamicin at 37 °C in 5% CO_2_. Cells were plated overnight at a density of 1 × 10^5^ cells/mL per well.

Twenty-four hours before the experiments, the medium was removed and replaced with a medium without phenol red supplemented with 5% dextran-coated, charcoal-treated FBS (5% DC-FBS) to exclude estrogenic effects caused by the medium. Next, cells were treated with selective antagonists: GPER [(3aS*,4R*,9bR*)-4-(6-bromo-1,3-benzodioxol-5-yl)-3a,4,5,9b-3*H*-cyclopenta[*c*]quinolone; G-15] (Tocris Bioscience, Bristol, UK), PPARα [*N*-((2*S*)-2-(((1*Z*)-1-methyl-3-oxo-3-(4-(trifluoromethyl)phenyl)prop-1-enyl)amino)-3-(4-(2-(5-methyl-2-phenyl-1,3-oxazol-4-yl)ethoxy)phenyl)propyl)propanamide, GW6471], or PPARγ [2-chloro-5-nitro-*N*-4-pyridinylbenzamide, T0070907] freshly prepared as stock solutions in dimethyl sulfoxide (DMSO) (Sigma-Aldrich) and stored at − 20 °C. Stock concentrations were subsequently dissolved in Waymouth’s media to a final concentration. Cells were treated with G-15, PPARα, or PPARγ alone or together for 24 h. Doses of G-15 (10 nM), PPARα (10 μM) or PPARγ (10 μM) (Gorowska-Wojtowicz et al. 2018). Control cells were treated with DMSO (final conc. 0.1%). Cell lysates and culture media were frozen in − 20 °C for further analyses.

### Scanning electron microscopy analysis

LCTs were fixed and processed with the use of Hitachi S-4700 scanning electron microscope (Hitachi, Tokyo, Japan) as previously described (Pawlicki et al. 2019).

### Histology

For routine histology, hematoxylin-eosin staining was performed on 4% paraformaldehyde LCT sections. As a control, commercially available paraffin sections of human testis (cat. no. HP-401; Zyagen, San Diego, CA, USA) were used.

### RNA isolation, reverse transcription, and real-time quantitative RT-PCR

Total RNA was extracted from LCTs specimens and commercially available normal human Leydig cells (cat. no 10HU-103; ixCells Biotechnologies, San Diego CA, USA) using TRIzol® reagent (Life Technologies, Gaithersburg, MD, USA) according to the manufacturer’s instructions. Total cDNA was prepared using High-Capacity cDNA Reverse Transcription Kit (Applied Biosystems, Carlsbad, CA, USA) according to the manufacturer’s instructions. A volume equivalent to 1 μg of total RNA was reverse transcribed. Total cDNA was prepared in a 20-μL volume using a random primer, dNTP mix, RNase inhibitor, and reverse transcriptase (RT). Parallel reactions for each RNA sample were run in the absence of RT to assess genomic DNA contamination. RNase-free water was added in place of the RT product.

RT-PCR was performed using the StepOne Real-Time PCR system (Applied Biosystems) and optimized standard conditions as described previously by Kotula-Balak et al. (2013, 2018b). Based on the gene sequences in Ensembl database, primer sets were designed using Primer3 software (Table 1). Selected primers were synthesized by Institute of Biochemistry and Biophysics, Polish Academy of Sciences (Warsaw, Poland). To calculate the amplification efficiency, serial cDNA dilution curves were produced for all genes. A graph of threshold cycle (Ct) versus log10 relative copy number of the sample from a dilution series was produced. The slope of the curve was used to determine the amplification efficiency: %E = (10^–1/slope^ − 1) × 100. All PCR assays displayed efficiency between 94 and 104%. Detection of amplification gene products was performed with 10 ng cDNA, 0.5 μM primers, and SYBR Green master mix (Applied Biosystems) in a final volume of 20 μL. Amplifications were performed as follows: 55 °C for 2 min, 94 °C for 10 min, followed by annealing temperature for 30 s (Table 1), and 45 s 72 °C to determine the cycle threshold (Ct) for quantitative measurement. To confirm amplification specificity, the PCR products from each primer pair were subjected to melting curve analysis and subsequent agarose gel electrophoresis (not shown). Images were captured using a Bio-Rad Gel Doc XR System (Bio–Rad Laboratories, Hercules, CA, USA) (not shown). mRNA expression within the control group was arbitrarily set as 1, against which statistical significance of experimental groups was analyzed. mRNA expressions were normalized to the *β-actin* mRNA (relative quantification, RQ = 1) with the use of the 2^−ΔΔCt^ method.

**Table 1:** Sequences of forward and reverse primers

Genes	Primers (5′–3′)	Product size (bp)	Annealing temperature (°C)	Cycles
*GPER*	5′-GCCTTGCAGTGGGGATGTCTCATAA-3′ 5′-GGATTCAGCTGGTCGATATCACTGGAG-3′	109	59.3	40
*PPARα*	5′-AGCCTCATGAAGAGCCTTCCAACTC-3′ 5′-CTGGATTCAGCTGGTCGATATCACTG-3	191	59.3	40
*PPARβ*	5′-CACTACGGTGTTCATGCATGTGAGG-3 5′-GTACTGGCACTTGTTGCGGTTCTTCT-3	129	59.3	40
*PPARγ*	5′-GATCAGCTCCGTGGATCTCTCCGTAA3′ 5′-GGAGATGCAGGCTCCACTTTGATTG-3′	207	61.1	40
*LHR*	5′-TGGCCTAGAGTCCATTCAGAGGCTAA-3 5′CAGCCAAATCAGGACCCTAAGGAAGT-3′	304	59.5	40
*PKA c.s. α*	5′-AACACAAGGAGACCGGGAACCACTAT-3′ 5′-CTCGAGTTTGACGAGGAACGGAAAG-3′	140	59.5	40
*PLIN 1*	5′-CTCCTCCCTCCAGACAAGGAAGAGTC-3′ 5′-TATCGAGAGAGGGTGTTGGTCAGAGC-3′	125	62.7	40
*HSL*	5′-AGCACTACAAACGCAACGAGACAGG-3′ 5′-GTTCTGTGTGATCCGCTCAAACTCAG-3′	119	59.3	40
*StAR*	5′-CTACAGTGACCAGGAGCTGGCCTATC-3′ 5′-CCCACATCTGGGACCACTTTACTCAT-3′	150	62.7	40
*TSPO*	5′-GTACGGCTCCTACCTGGTCTGGAAAG-3′ 5′-ACGCAGTAGTTGAGTGTGGTCGTGAA-3′	279	62.7	40
*HMGCS1*	5′-CTCTTTCACCATGCCTGGATCACTTC-3′ 5′-GCATTTGGCCCAATTAGCAGAGCTAC-3′	564	59.5	40
*HMGCR*	5′GCTCTCCTTCTGGCTGTCAAGTACATC-3′ 5′-CTCCTTTATCACTGCGAACCCTTCAG-3′	343	61.3	40
*β-actin*	5′-AGTTGCGTTACACCCTTTCTTG-3′ 5′-CACCTTCACCGTTCCAGTTTT-3′	235	61	40

*GPER* G-coupled estrogen receptor, *PPARα* peroxisome proliferator–activated receptor alpha, *PPARβ* peroxisome proliferator–activated receptor beta, *PPARγ* peroxisome proliferator–activated receptor gamma, *LHR* lutropin receptor, *PKA c.s.* α protein kinase catalytic subunit alpha, *PLIN1* perilipin 1, *HSL* hormone sensitive lipase, *StAR* steroidogenic acute regulatory protein, *TSPO* translocator protein, *HMGCS* 3-hydroxy-3-methylglutaryl-CoA synthase 1, *HMGCR* 3-hydroxy-3-methylglutaryl-CoA reductase, *β-actin* beta-actin

### Western blotting

For quantification of protein expression (Table 2), LCT proteins (as a control commercially available normal human Leydig cells; cat. No 10HU-103; ixCells Biotechnologies, San Diego CA, USA) were extracted in radioimmunoprecipitation assay buffer (RIPA; Thermo Scientific, Inc. Rockford IL, USA). Aliquots (50 μg protein) of cell lysates were used for electrophoresis on 12% mini gel by standard SDS-PAGE procedures and electrotransferred to polyvinylidene difluoride (PVDF) membranes (Millipore Corporate, MA, USA) by a semi-dry transfer cell (Bio-Rad). Then, blots were blocked with 5% non-fat dry milk in TBS, 0.1% Tween 20, overnight at 4 °C with shaking, followed by an incubation with respective antibodies (Table 2).

**Table 2:** Primary antibodies used for immunohistochemistry and Western blot

Antibody	Host species	Vendor	Dilution
GPER	Rabbit	Abcam cat. no. 39742	1:50 (IHC) 1:2000 (WB)
PPARα	Mouse	Thermo Fisher Scientific cat. no. MA1–822	1:50 (IHC) 1:1000 (WB)
PPARβ	Rabbit	Thermo Fisher Scientific Cat. No. PA1-823A	1:250 (IHC) 1:2000 (WB)
PPARγ	Rabbit	Abcam cat. no. 209350	1:100 (IHC) 1:4000 (WB)
LHR (H-50)	Rabbit	Santa Cruz Biotechnology cat. no. sc-25,828	1:20 (IHC) 1:1000 (WB)
PKAIIα	Rabbit	Santa Cruz Biotechnology cat. no. sc-908	1:200 (IHC) 1:5000 (WB)
PLIN-1	Rabbit	Cell Signaling Technology cat. no. 9349	1:50 (IHC) 1:2000 (WB)
HSL	Rabbit	Cell Signaling Technology cat. no. 4107	1:100 (IHC) 1:1000 (WB)
StAR	Mouse	Abcam cat. no. ab5813	1:100 (IHC) 1:1000 (WB)
TSPO	Goat	Thermo Fisher Scientific cat.no. PA5–18565	1:100 (IHC) 1:1000 (WB)
HMGCS1	Rabbit	Abcam cat. no. ab155787	1:250 (IHC) 1:2000 (WB)
HMGCR	Rabbit	Thermo Fisher Scientific cat.no. PA5–18565	1:50 (IHC) 1:1000 (WB)
PI3Kp85	Rabbit	Cell Signaling Technology cat. no. 4292	1:500 (WB)
t-Akt	Rabbit	Cell Signaling Technology cat. no. 9272S	1:1000 (WB)
mTOR	Rabbit	Cell Signaling Technology cat. no. 9272S	1:1000 (WB)
β-actin	Mouse	Sigma–Aldrich cat. no. A2228	1:3000 (WB)

*GPER* G-coupled estrogen receptor, *PPARα* peroxisome proliferator–activated receptor alpha, *PPARβ* peroxisome proliferator–activated receptor beta, *PPARγ* peroxisome proliferator–activated receptor gamma, *LHR* lutropin receptor, *PKA c.s.* α protein kinase catalytic subunit alpha, *PLIN1* perilipin 1, *HSL* hormone sensitive lipase, *StAR* steroidogenic acute regulatory protein, *TSPO* translocator protein, *HMGCS* 3-hydroxy-3-methylglutaryl-CoA synthase 1, *HMGCR* 3-hydroxy-3-methylglutaryl-CoA reductase, *PI3K* phosphatidylinositol-45-bisphosphate 3-kinase, *t-Akt* Akt-serine/threonine-specific protein kinase (protein kinase B), *mTOR* the mammalian target of rapamycin kinase

The membranes were washed and incubated with a secondary antibody conjugated with the horseradish-peroxidase labeled goat anti-mouse or goat anti-rabbit IgGs (Vector Labs., Burlingame, CA, USA) at a dilution 1:1000, for 1 h at RT. Immunoreactive proteins were detected by chemiluminescence with Western Blotting Luminol Reagent (Santa Cruz Biotechnology), and images were captured with a ChemiDoc XRS + System (Bio-Rad Laboratories). All immunoblots were stripped with stripping buffer containing 62.5 mM Tris–HCl, 100 mM 2-mercaptoethanol, and 2% SDS (pH 6.7) at 50 °C for 30 min and incubated in a mouse monoclonal antibody against β-actin. Each data point was normalized against its corresponding β-actin data point.

Quantitative analysis was performed for three separately repeated experiments using a public domain ImageJ software (National Institutes of Health, Bethesda, MD) as described elsewhere (Smolen 1999). Protein level within the control group was arbitrarily set as 1, against which statistical significance of experimental groups was analyzed. The relative protein levels were expressed as arbitrary units.

### Immunohistochemistry

To optimize immunohistochemical staining testis sections, both commercially available control (Zyagen, San Diego, CA, USA) and LCT sections were immersed in 10 mM citrate buffer (pH 6.0) and heated in a microwave oven (2 × 5 min, 700 W). After overnight incubation at 4 °C with primary antibodies, (they are listed in Table 2) respective biotinylated antibodies (anti-rabbit and anti-mouse IgGs; 1: 400; Vector, Burlingame CA, USA) and avidin-biotinylated horseradish peroxidase complex (ABC/HRP; 1:100; Dako, Glostrup, Denmark) were applied in succession. Bound antibody was visualized with 3,3′-diaminobenzidine (DAB) (0.05%; v/v; Sigma-Aldrich) as a chromogenic substrate. Control sections included omission of primary antibody and substitution by irrelevant IgG (Bilinska et al. 2018).

### cGMP concentration and estradiol secretion

The production of cGMP in control and treated with GPER and PPAR antagonists (alone or in combinations) MA-10 cells was measured by General Cyclic guanosine monophosphate ELISA kit assay (EIAab Wuhan Eiaab Science Co., LTD, Wuhan, China) according to the manufacturer’s instructions with detection level 0.31 to 20.0 ng/mL. The cGMP levels were calculated as nanograms per milliliter.

Estradiol Enzyme Immunoassay Kit (DRG, Inc. Int. Springfield, USA) was used for measurement of estradiol content in culture medium from control and treated with GPER and PPAR antagonists (alone or in combinations) MA-10 cells according to the manufacturer’s instructions. The sensitivity of the assay was 10.6 pg/mL. The absorbance (*λ* = 450 nm) was measured. Data were expressed as mean ± SD.

The measurements were performed with the use ELISA apparatus (Labtech LT-4500).

### Statistics

Three biological repeats of each sample (*n* = 7) and three independent experiments were performed. Each variable was tested using the Shapiro-Wilk *W* test for normality. The homogeneity of variance was assessed with Levene’s test. Comparisons were performed by one-way ANOVA, followed by Dunnett’s post hoc test (GB-STAT software, v. 7.0; Dynamic Microsystems) to determine the significant differences between proteins expression levels, cGMP content, and estradiol secretion. Statistical analyses were performed on raw data using Statistica 10 software (StatSoft Inc., Tulsa, OK, USA)*.* Data were presented as means *±* S.D. Data were considered statistically significant at ∗*p* < 0.05, ∗∗*p* < 0.01, and ∗∗∗*p* < 0.001.

## Results

### Scanning electron microscopic and morphological observations of LCTs

**Fig. 1:**
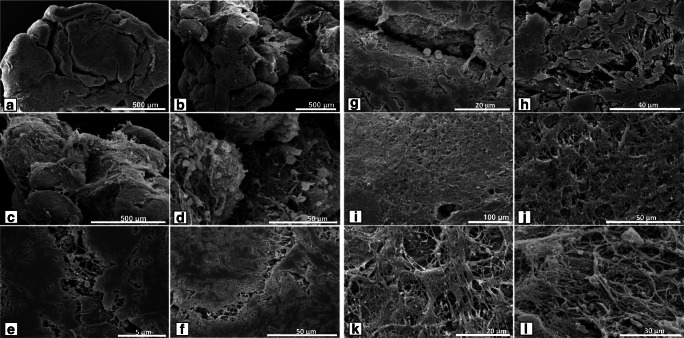
Morphology of human Leydig cell tumors—scanning electron microscopic analysis (**a** A–F and **b** G–L). (A–D) General structure of fragments of testicular biopsies used for analysis. (E, F) Fragments of tissue with tumor. Note solid tumor structure with areas separated by deep grooves. (E, F) and **b** (G, H) cellular masses infiltrating each other. (I–L) Tumor Leydig cells in mass tightly linked by thick projections formed a cage-like structure covering individual tumor sheets. Representative microphotographs of scanning electron microscopic analysis of human Leydig cell tumors (LCTs). Bars represent 1 μm. For analysis, *n* = 12 specimens were used

Scanning electron microscopy analyses of LCT biopsy fragments (Fig. 1 A–D) revealed the tumors were relatively compact structures of oval or slightly elongated shape (Fig. 1 A–C) with tumor cells apposed and tightly adhering to one another (Fig. 1 E, F).

It is important to note that some tumor cells were fused. In addition, compact areas of tumor cells were separated by deep grooves. Between those grooves, compact tumor sheets were formed (Fig. 1 E–G). Cells within sheets were compactly linked by thick projections and masses of such connections were observed between cells from neighboring tumor sheets (Fig. 1 E, F). Higher magnification revealed the presence of elongated, delicate filiform fibrillar projections that formed a cage-like structure covering individual tumor sheets (Fig. 1 I–L).

**Fig. 2:**
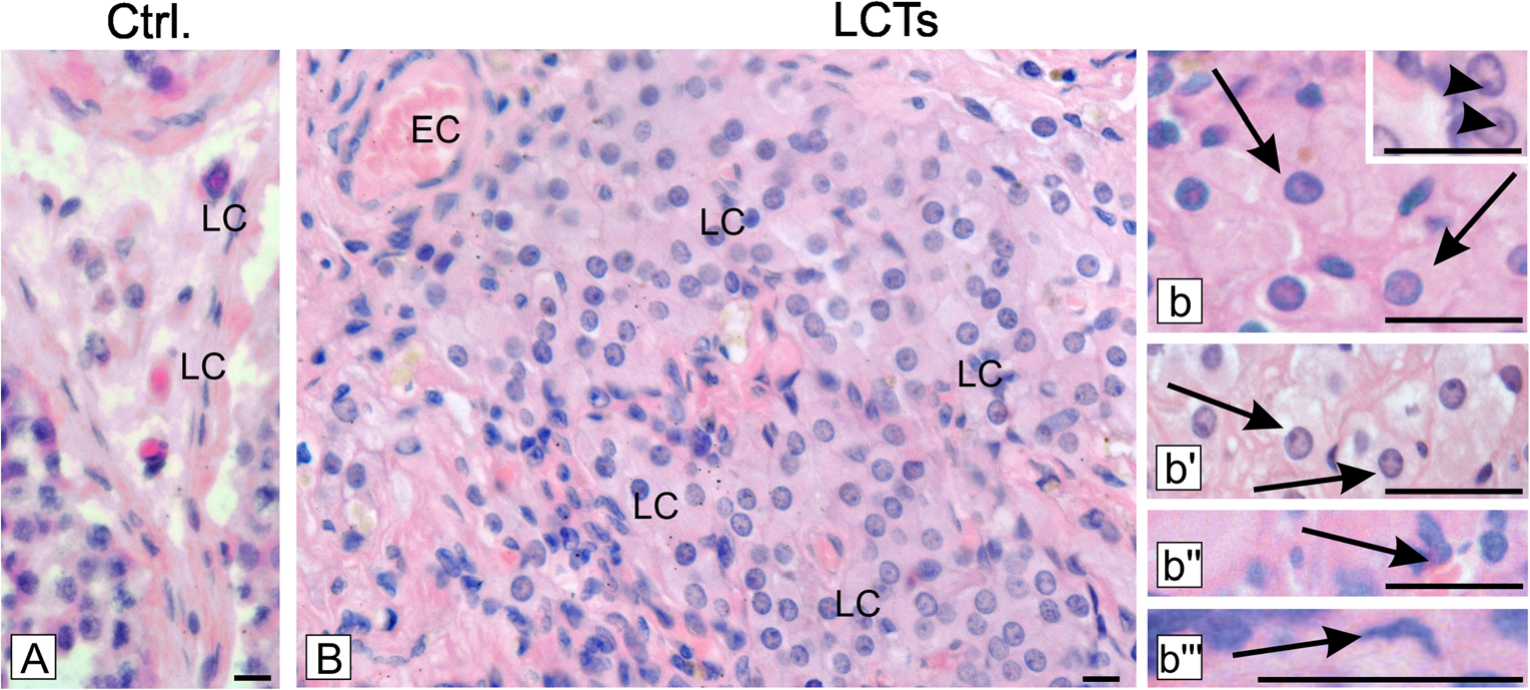
Morphology of human Leydig cell tumors—hematoxylin-eosin staining representative microphotographs of (A) control human testis and (B, b–b”’) Leydig cell tumors (LCTs). Scale bars represent 30 μm. Staining was performed on serial testicular sections from *n* = 12 specimens. LC - Leydig cells; EC - epithelial cells of blood vessels. (b)— cells of large polygonal shape with abundant, cytoplasm, indistinct cell borders, and regular, round to oval nuclei (arrows). Prominent nucleus visible at (b) higher magnification (arrowheads), (b’)— cells with above features but possessing distinct cell borders and smaller nuclei (arrows), (b”)— small cells with scant, densely eosinophilic cytoplasm and grooved nuclei (arrow), (b”’)— spindle-shaped (sarcomatoid) cells (arrow)

Hematoxylin-eosin staining demonstrated a mixture of four morphological types of cells in the tumor mass when compared to controls, where single or small groups of Leydig cells were seen in the interstitial space (Fig. 2 A, B).

In LCTs, most cells possessed a large polygonal shape with abundant cytoplasm, indistinct cell borders, and regular round to oval nuclei. The nucleus was found to be frequently prominent (Fig. 2 b). Occasionally, cells as those noted above were found to possess distinct cell borders and smaller nuclei (Fig. 2 b’). Small cells with scant, densely eosinophilic cytoplasm and a grooved nucleus (Fig. 2 b”) and spindle-shaped (sarcomatoid) cells (Fig. 2 b”’) were observed as well.

### Expression and localization of GPER and PPARs in LCTs

**Fig. 3:**
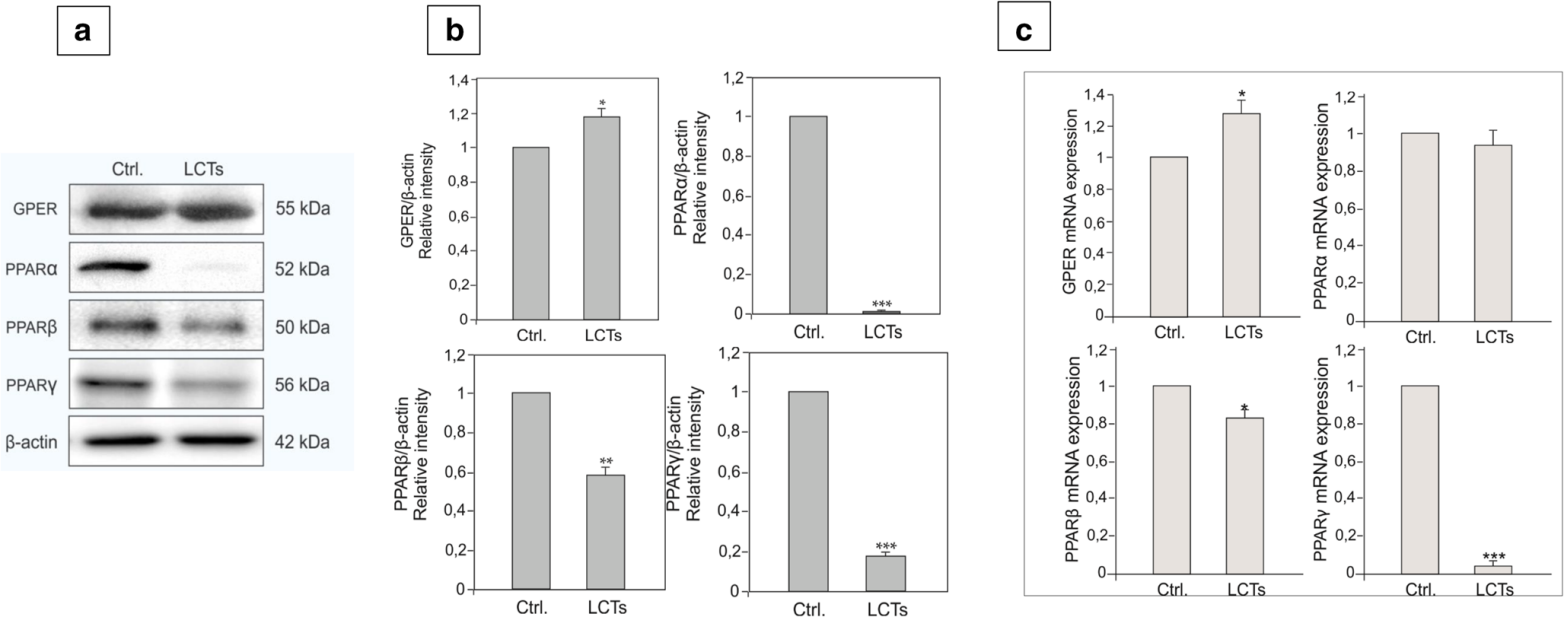
Expression of GPER, PPARα, PPARβ, and PPARγ in human Leydig cell tumor. (**a**) Representative immunoblots of qualitative expression of GPER, PPARα, and PPARγ and (**b**) relative expression (quantitative representation after densitometry of data; arbitrary units). The relative amount of respective proteins normalized to β-actin. Relative intensity from three separate analyses is expressed as means ± SD. Asterisks show significant differences between respective control and Leydig cell tumor (LCTs). Values are denoted as ∗*p* < 0.05, ∗∗*p* < 0.01, and ∗∗∗*p* < 0.001. Analysis was performed in triplicate (*n* = 7). (**c**) Relative level (relative quantification; RQ) of mRNA for GPER, PPARα, PPARβ, and PPARγ determined using real-time RT-PCR analysis 2−ΔCt method. As an intrinsic control, *β-actin* mRNA level was measured in the samples. RQ from three separate analyses is expressed as means ± SD. Asterisks show significant differences between respective control and Leydig cell tumor (LCTs). Values are denoted as ∗*p* < 0.05 and ∗∗∗*p* < 0.001. Analysis was performed in triplicate (*n* = 7)

In LCTs, increased expression of GPER (*p* < 0.05) and decreased expression of PPARα (*p* < 0.001), PPARβ (*p* < 0.01), and PPARγ (*p* < 0.001) were seen when compared to controls (Fig. 3 a, b). Corresponding to GPER and PPAR protein expressions, changes in their mRNA expressions in LCTs were found (Fig. 3).

**Fig. 4:**
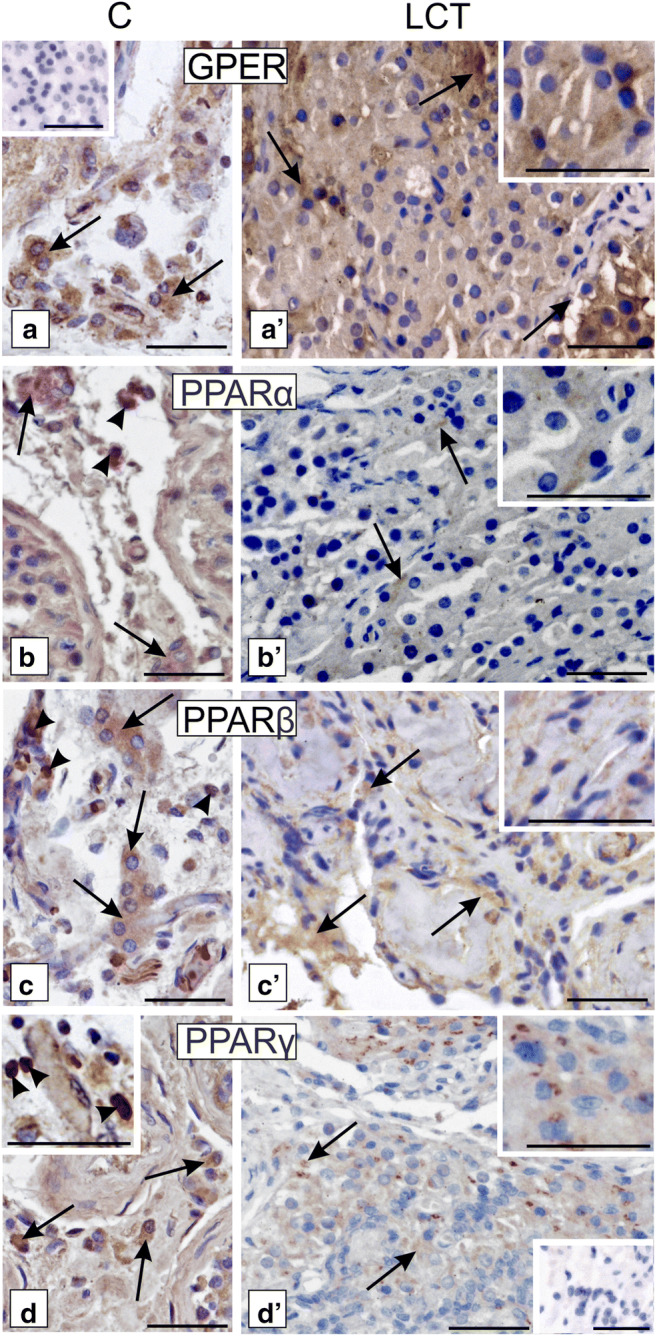
Localization of GPER, PPARα, PPARβ, and PPARγ in human Leydig cell tumor. Representative microphotographs of cellular localization of GPER (a, a’ and higher magnification at a’), PPARα (b, b’ and higher magnification at b’), PPARβ (c, c’ and higher magnification at c’), and PPARγ (d, d’ and higher magnification at d and d’) in control human testes (a–d and higher magnification at d) and Leydig cell tumor (LCTs). DAB immunostaining with hematoxylin counterstaining. Scale bars represent 100 μm. Staining was performed on serial testicular sections from *n* = 12 specimens. Arrows depict cytoplasmic staining; arrowheads depict nuclear staining. No positive staining is seen when the primary antibodies were omitted—insert at a and d’ (negative controls)

No changes in GPER localization and staining intensity were found in control Leydig cells and LCTs (Fig. 4 a, a’).

Specifically, the staining was exclusively cytoplasmic and of moderate intensity. Localization and immunostaining intensity of PPAR varied between Leydig cells of control testis and LCTs (Fig. 4 b, b’, c, c’, d, d’). While strong cytoplasmic-nuclear expression of PPARα, β, and γ was found in control samples, weak PPARα immunoexpression and moderate-to-very weak expression of PPARβ and PPARγ, respectively, were detected. In LCTs, PPARs were located primarily in the cytoplasm of Leydig cells. No positive staining was found when primary antibodies were omitted (Fig. 4, inserts at a, d’).

### Expression and localization of LHR, PKA, PLIN, HSL, StAR, TSPO, HMGCS, and HMGCR in LCTs

**Fig. 5:**
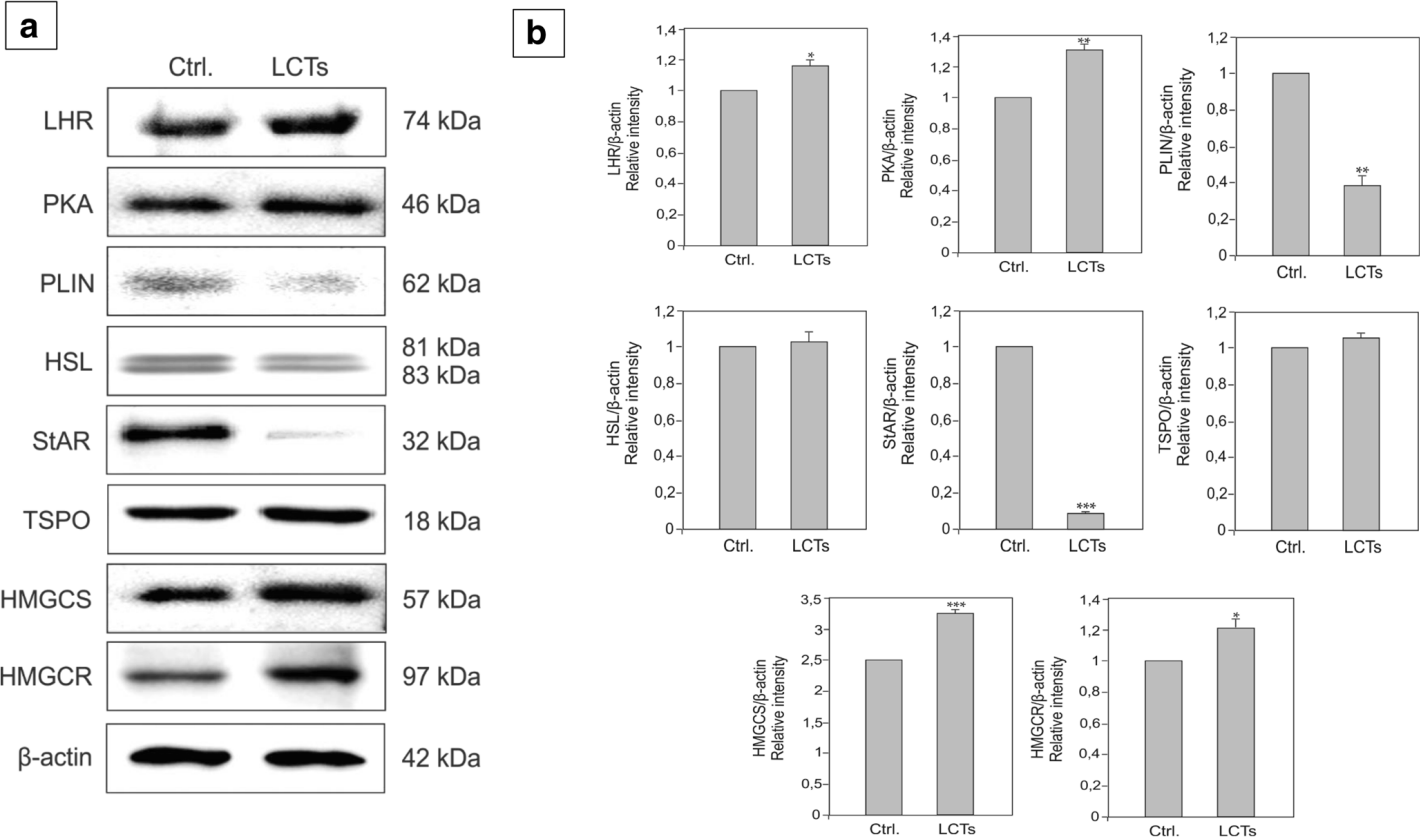
Expression of LHR, PKA, PLIN, HSL, StAR, TSPO, HMGCS, and HMGCR in human Leydig cell tumor. (**a**) Representative blots of qualitative expression of LHR, PKA, PLIN, HSL, PLIN, StAR, TSPO, HMGCS, and HMGCR and (**b**) relative expression (quantitative representation after densitometry of data; arbitrary units). The relative amount of respective proteins normalized to β-actin. Relative intensity from three separate analyses is expressed as means ± SD. Asterisks show significant differences between respective control and Leydig cell tumor (LCTs). Values are denoted as ∗ *p* < 0.05, ∗∗ *p* < 0.01, and ∗∗∗*p* < 0.001. Analysis was performed in triplicate (*n* = 7)

In LCTs, varied expression of LHR, PKA, PLIN, HSL, StAR, TSPO, HMGCS, and HMGCR was observed when compared to normal Leydig cells (Fig. 5 a, b).

**Fig. 6:**
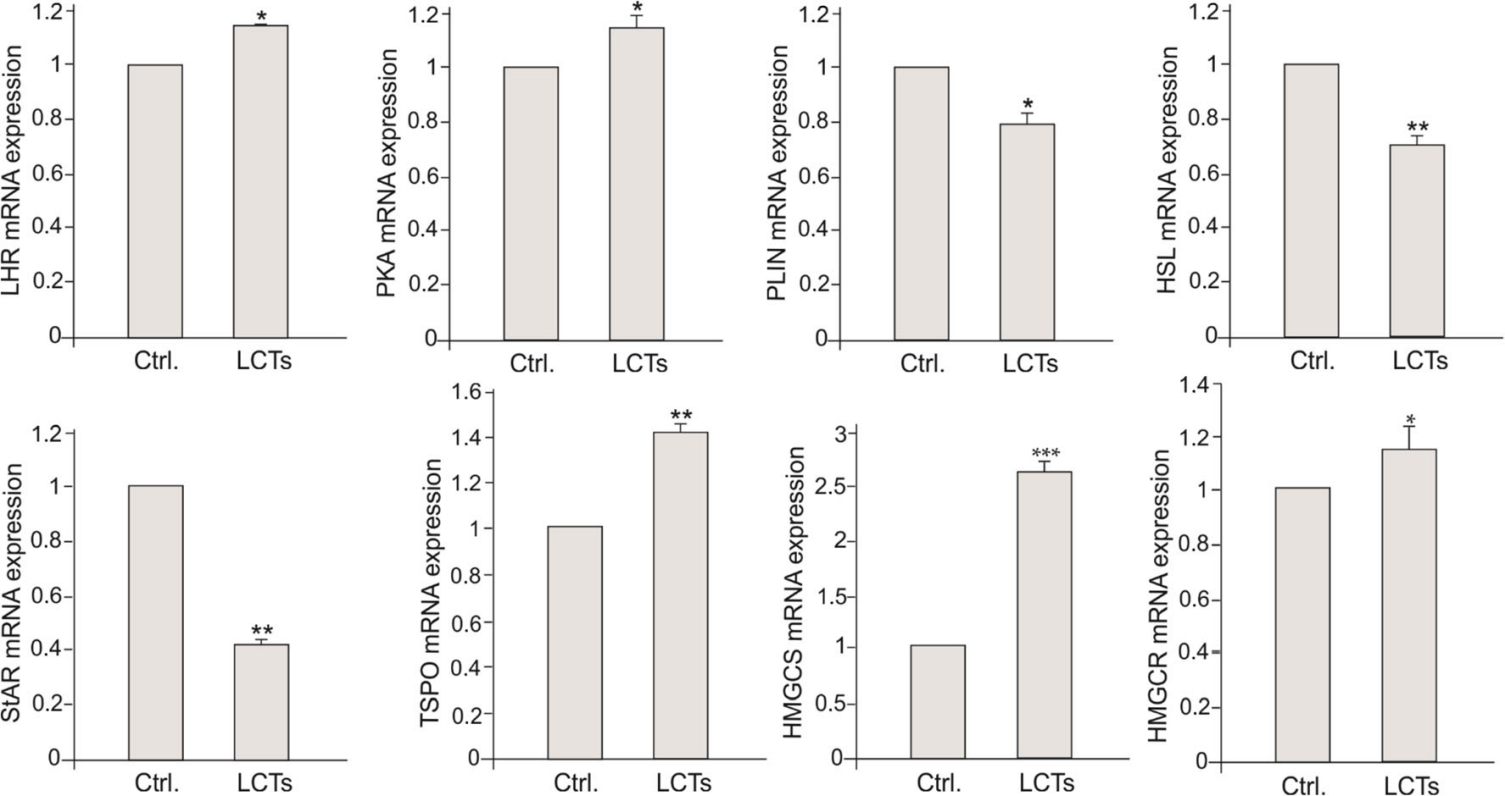
Expression of mRNA for *LHR*, *PKA*, *PLIN*, *HSL*, *StAR*, *TSPO*, *HMGCS*, and *HMGCR* in human Leydig cell tumor. Relative level (relative quantification; RQ) of mRNA for *LHR*, *PKA*, *PLIN*, *HSL*, *PLIN*, *StAR*, *TSPO*, *HMGCS*, and *HMGCR* determined using real-time RT-PCR analysis 2−ΔCt method. As an intrinsic control, *β-actin* mRNA level was measured in the samples. RQ from three separate analyses is expressed as means ± SD. Asterisks show significant differences between respective control and Leydig cell tumor (LCTs). Values are denoted as ∗*p* < 0.05, ∗∗*p* < 0.01, and ∗∗∗*p* < 0.001. Analysis was performed in triplicate (*n* = 7)

The expression of LHR and PKA was increased (*p* < 0.05 and *p* < 0.01, respectively) as well as that of HMGCS and HMGCR (*p* < 0.001 and *p* < 0.05, respectively). In contrast, PLIN and StAR expressions were decreased (*p* < 0.001 and *p* < 0.05, respectively), while a non-significant increase was observed for HSL and TSPO. Changes were also found for mRNA LHR, PKA, PLIN, HSL, StAR, TSPO, HMGCS, and HMGCR expressions in LCTs (Fig. 6).

**Fig. 7:**
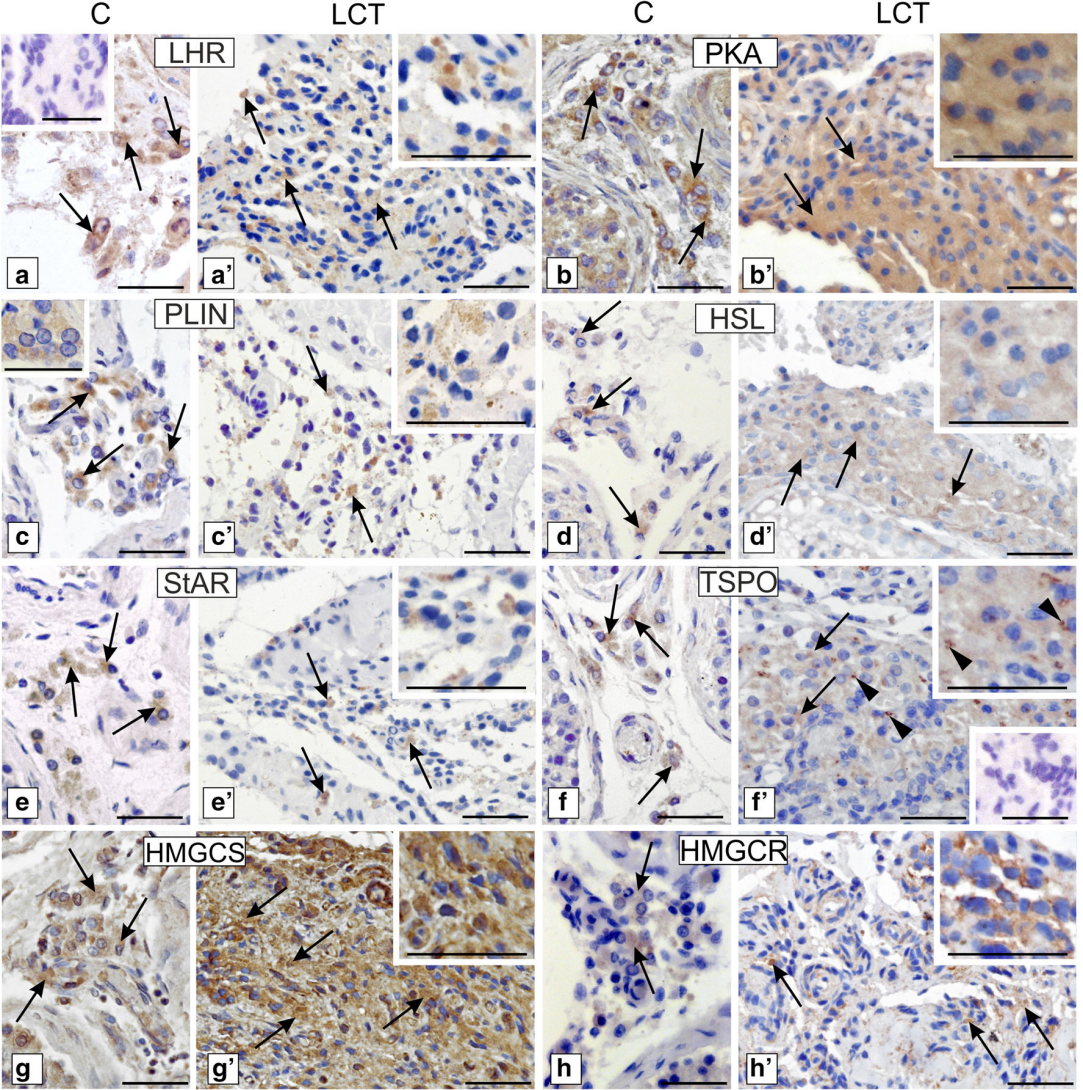
Localization of LHR, PKA, PLIN, HSL, StAR, TSPO, HMGCS, and HMGCR in human Leydig cell tumor. Representative microphotographs of cellular localization of LHR (a–a’), PKA (b–b’), PLIN (c–c’ and higher magnifications at c and c’), HSL (d–d’), StAR (e–e’), TSPO (f–f’), HMGCS (g–g’), and HMGCR (h–h’) in control human testes (a–h and higher magnification at c) and Leydig cell tumor (a’–h’ and higher magnification at c’). DAB immunostaining with hematoxylin counterstaining. Scale bars represent 100 μm. Staining was performed on serial testicular sections from *n* = 12 specimens. Arrows depict cytoplasmic staining. Arrowheads depict strong stained cells for TSPO and positively stained epithelial cells of blood vessels for HSL. No positive staining is seen when the primary antibodies were omitted—insert at a and f’ (negative controls)

In control Leydig cells, membrane cytoplasmic and LCT cytoplasmic stainings of LHR were found (Fig. 7 a, a’).

The immunostaining was of moderate intensity in control Leydig cells but was weak and present in minority of cells of LCTs. No differences were found in PKA distribution and immunostaining (Fig. 7 b, b’), with strong staining present in control and tumor Leydig cell cytoplasm. PLIN distribution, reflecting distribution of lipid droplets, was cytoplasmic in control Leydig cells and LCTs (Fig. 7 c, c’). In control Leydig cells, staining intensity was strong while found to be weak in LCTs. Increased HSL staining intensity was detected in LCTs when compared to control cells (Fig. 7 d, d’) and was exclusively cytoplasmic. Strong immunoreaction was found in the blood vessel epithelium. In contrast, decreased staining intensity of StAR, exclusively present in the cytoplasm (as diffuse signal indicating on distribution of mitochondria), was observed in a few cells of LCTs (Fig. 7 e, e’) while control Leydig cells exhibited moderate cytoplasmic staining. A similar pattern was found for mitochondrial TSPO (Fig. 7 f, f’). Moderate, diffused cytoplasmic expression was revealed in control Leydig cell cytoplasm, while the TSPO staining intensity was very weak but still diffused in LCTs. However, in a few cells, immunoreaction was very strong. No differences were found in the distribution of HMGCS and HMGCR between control cells and LCTs (Fig. 7 g, g’ and h, h’). Strong cytoplasmic expression of HMGCS and moderate cytoplasmic expression of HMGCR were revealed in control Leydig cells and LCTs, respectively. No positive staining was found when primary antibodies were omitted (Fig. 7, inserts at a, f’).

### Effect of GPER and PPAR blockage on expression of PI3K, Akt, and mTOR in LCTs and mTOR in MA-10 cells

**Fig. 8:**
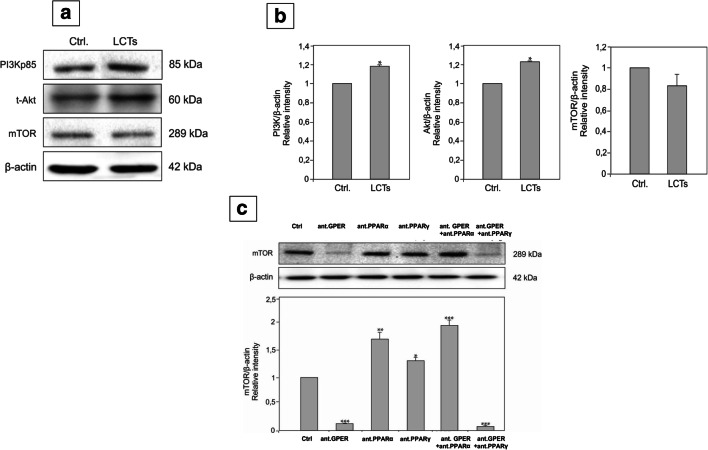
Expression of PI3K-Akt-mTOR pathway in human Leydig cell tumor. (**a**) Representative blots of qualitative expression of PI3K, Akt, mTOR, and (**b**) relative expression (quantitative representative after densitometry of data; arbitrary units). The relative amount of respective proteins normalized to β-actin. Relative intensity from three separate analyses is expressed as means ± SD. Asterisks show significant differences between control and Leydig cell tumor (LCTs). Values are denoted as ∗*p* < 0.05. Analysis was performed in triplicate (*n* = 7). (**c**) Effect of GPER and PPAR blockage on expression of mTOR in MA-10 cells. Representative blots of qualitative expression of mTOR and relative expression (quantitative representation after densitometry of data; arbitrary units). The relative amount of protein normalized to β-actin. Relative intensity from three separate analyses is expressed as means ± SD. Asterisks show significant differences between control and treated Leydig cells. Values are denoted as ∗*p* < 0.05, ∗∗*p* < 0.01, and ∗∗∗*p* < 0.001. Analysis was performed in triplicate (*n* = 3 for each experimental group)

In LCTs, PI3K and Akt expressions were increased (*p* < 0.05) while no changes in mTOR expression were found when compared to controls (Fig. 8 a, b).

In MA-10 cells, expression of mTOR was decreased (*p* < 0.001) after blockage of GPER or GPER together with PPARγ (Fig. 8 c). Blockage of PPARα, PPARγ, or GPER together with PPARγ increased (*p* < 0.05, *p* < 0.01, *p* < 0.001) mTOR expression.

### Effect of GPER and PPAR blockage on estradiol secretion and cGMP concentration in MA-10 cells

**Fig. 9:**
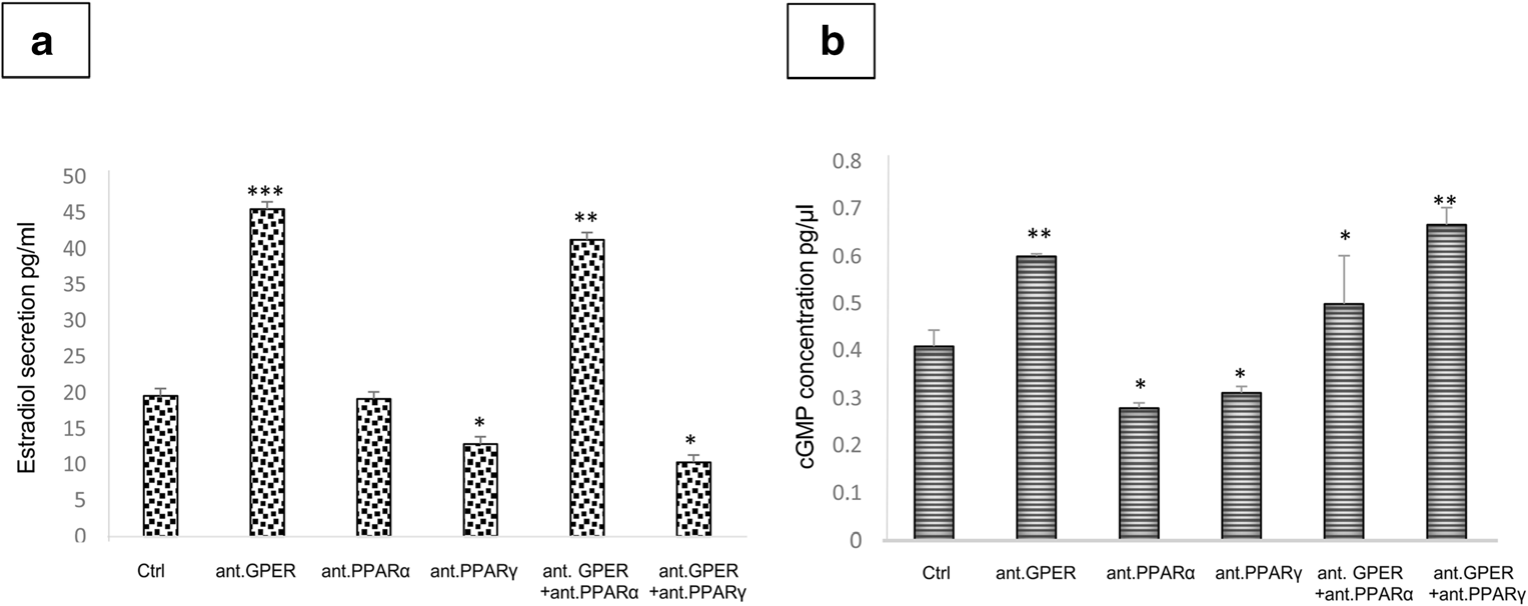
Effect of GPER and PPAR blockage on expression on estradiol secretion and cGMP concentration in MA-10 cells. Estradiol secretion (**a**) and cGMP concentration (**b**) in control and treated with GPER (10 nM), PPARα (10 μM) and PPARγ (μM) antagonists alone or in combinations for 24 h tumor mouse Leydig cells (MA-10). Asterisks show significant differences between control and treated Leydig cells. Values are denoted as ∗*p* < 0.05, ∗∗*p* < 0.01, and ∗∗∗*p* < 0.001. Analysis was performed in triplicate (*n* = 3 for each experimental group)

Secretion of estradiol markedly increased (*p* < 0.001) after GPER blockage (Fig. 9 a). A similar increase (*p* < 0.01) was observed after GPER and PPARα blockage. Conversely, blockage of GPER and PPARγ decreased (*p* < 0.05) estradiol secretion. When either PPARα or PPARγ was blocked, no to little alterations (*p* < 0.05) in hormone secretion were revealed.

Changes in cGMP concentration after antagonist-treatment were similar to those of estradiol secretion (Fig. 9 b). Treatment with a GPER antagonist, alone or in combination with a PPARα antagonist, increased (*p* < 0.05, *p* < 0.01) cGMP concentration while treatment with PPARα or γ antagonists consistently decreased (*p* < 0.05) the concentration. Only treatment with GPER and PPARγ antagonists in combination increased (*p* < 0.01) cGMP concentration.

## Discussion

Benign LCTs are classically presented as a small (3–5 cm in diameter), sharply delineated, and solid mass embedded within the testis (Al-Agha and Axiotis 2007). Alternatively, malignant LCTs are typically larger (greater than 5 cm in diameter), have infiltrative margins, and show areas of hemorrhage and necrosis. They replace the testis and/or extend beyond testicular parenchyma. For the first time, we showed by scanning electron microscope a complicated LCT cellular organization. We found that individual cells were not recognized in the solid mass, but a number of prolongations of various sizes were formed, maintaining cells tightly linked to each other; however, this did not let to distinguish benign and malignant tumors. Morphologically, LCTs can consist predominantly of one type or as a mixture of the four types of cells with abundant lipid accumulation (Richmond et al. 1995). This is in agreement with our observations. In addition, our immunohistochemical results of StAR and PLIN may suggest that LCT cells are overloaded with lipids that probably are not processed to mitochondria.

A central factor in LCT growth and progression is represented by an inadequate intratesticular balance in the androgen/estrogen ratio with advantage of the latter hormone (Carreau et al. 2010; Fowler et al. 2000; Sirianni et al. 2007). Varying expression patterns of ERα and ERβ were observed in human LCTs compared to healthy testis (Carpino et al. 2007). Also, in human and rat LCTs, involvement of GPER in cell proliferation, growth, and apoptosis was shown. Rago et al. (2009, 2011) confirmed the presence of GPER in germ cell tumors and sex-cord stromal tumors. However in LCTs, the authors found no differences in GPER expression in relation to normal testis. In contrast, herein, we revealed an increase in GPER expression in LCTs. Also in our in vitro experiments in mouse tumor Leydig cells, GPER expression was increased (Gorowska-Wojtowicz et al. 2018). Taken together, it is likely that various estrogen pathways may be deregulated in LCTs, which reflects tumor heterogeneity and may contribute to its development.

Herein, we showed GPER, alone and together with PPARα, effected estradiol secretion by tumor Leydig cells. Such result indicates on a leading role of GPER in regulation of sex hormone production and secretion and concomitantly suggests possible GPER and PPARα alterations in LCTs. Similarly, our prior study also showed progesterone secretion modulation in GPER and PPAR antagonist-treated tumor mouse Leydig cells (Gorowska-Wojtowicz et al. 2018). According to findings by Chimento et al. (2011), GPER is a good target for reduction of tumor Leydig cell proliferation that is hormonally controlled.

We showed, for the first time, a PPAR expression pattern in normal human Leydig cells and its prominent downregulation in LCT. An opposite correlation was found in dog testis, and PPAR expression was always markedly higher in tumor tissue (Sozmen et al. 2013). Notably, confusing results were seen concerning the involvement of PPAR in tumor biology. PPAR was revealed to both promote and inhibit cancer via effects on cell differentiation, growth, metastasis, and lipid metabolism (Maan et al. 2018).

In our findings, both in vivo and in vitro studies revealed possible relationship between GPER, PPAR, and lipid homeostasis-controlling molecules in LCT. Recent studies have linked lipid abundance with increased tumor aggressiveness and its resistance to chemotherapy (Tirinato et al. 2017). Findings by Christian et al. (2013) showed that autophagy influences lipid metabolism via both lipogenesis and lipolysis. Lipid droplets may induce lipophagy to avoid lipotoxicity, a phenomenon caused by excessive lipid accumulation with involvement of the mTOR signaling pathway (Yang et al. 2018). Increased cholesterol content without activated mTOR can be an indirect result of lipophagy and/or PLIN alterations in LCTs. It seems this particular tumor can have a distinct biology, but it is possible that some mechanisms may be induced later when its development is more advanced. Last data demonstrate that lipophagy contributes to testosterone biosynthesis at least partially through degrading lipid droplets and cholesterol content (Ma et al. 2018). In steroidogenic cells, the mechanism underlying lipid turnover and receptor involvement remained unrevealed (Maizlin et al. 2004); however, based on our results, GPER-PPAR cross-talk should be taken into consideration.

We found prominent changes in LHR, StAR, and PKA expressions, reflecting disturbances in lipid controlling mechanisms directly associated with central endocrine regulation and the local microenvironment. In LCT, we detected decreased expression of StAR and increased expression of LHR. In contrary, in tumor mouse Leydig cell line (mLTC-1), StAR activity was increased while LHR expression was significantly reduced (Manna et al. 2007). These discrepancies may be due to species specific characteristics of tumor Leydig cells or may be ascribed to above-mentioned tumor heterogeneity or complex mechanisms regulating protein biology (Bauer et al. 2015). Nevertheless, the regulation of steroidogenesis is clearly disturbed in LCT. Moreover, in our study, LHR was found in the cytoplasm as this receptor can be internalized. Internalization is a part of the physiological mechanism of action for many G-coupled receptors in a specific condition, e.g., agonist treatment, influence of tissue/cell microenvironment (Reubi 2014). In pathological conditions, translocation of proteins is usually observed phenomenon.

Perturbations in StAR expression patterns observed in the present study may reflect altered mitochondrial function and/or degradation (mitophagy), thus affecting lipid homoeostasis in TLCs (Barbosa and Siniossoglou 2017). Herein, HSL and TSPO expressions did not vary in LCTs, suggesting a subordinate role of these molecules in LCTs.

Lipid droplet associated proteins are actively involved in modulating lipid homeostasis by generating sites for steroidogenic enzyme activity (Shen et al. 2016). Changes in PLIN expression found in our study may suggest that in LCT not only transport of cholesterol to mitochondria but also lipid storage and mobilization from lipid droplets are dysregulated. This may be considered as additional factor aiding in the development of LCTs.

Enhanced expression of sterol regulatory element binding proteins (SREBPs), involved in cholesterol and fatty acids synthesis through the Akt pathway (anchored-lipid membrane protein), correlates with tumor development, progression, and invasiveness, as well as increased lipid content in cell membranes (Beloribi-Djefaflia et al. 2016). Through both the post-translational regulation and induction of transcriptional programs, the dysregulated PI3K-Akt-mTOR pathway coordinates the uptake and utilization of multiple nutrient lipids supporting the enhanced growth and proliferation of cancer cells (Basharat et al. 2016). In this study, we found PI3K, Akt, and mTOR signaling alterations, which indicate that also LCT deregulation of these pathways may be an important event accompanying tumor development. Notably, changes in hormonal milieu including estradiol can result in modulation and mTOR activity (Blagosklonny 2010). Our earlier studies in mouse tumor Leydig cells revealed that GPER and PPAR inhibitions activated PI3K and Akt (Gorowska-Wojtowicz et al. 2018). Here we showed that in addition, mTOR was modulated diversely dependently on receptor antagonist used alone or in combination, inhibited by GPER antagonist alone and together with PPARγ antagonist as well as activated by GPER with PPARα together and the latter alone. Therefore, these results demonstrated that GPER- and PPARα-mediated pathways are involved in the maintenance of mTOR activity, whereas PPARγ signaling has an opposite effect, reducing mTOR activity.

Distinct changes in cGMP level suggest GPER-PPAR–mediated high or only via PPAR low metastatic activity of LCT. cGMP agonist was confirmed as a mediator of lipolysis in oocytes (Mendoza et al. 2011). In LCTs, cGMP seems to be important in maintaining lipid homeostasis when GPER and PPAR are absent.

The outcome of lipid content modification is a result of various protein–protein cross-talk that was revealed here also between GPER-PPAR and HMGCS and HMGCR resulting in overexpression of the latter enzymes that is in line with our earlier in vitro study (Gorowska-Wojtowicz et al. 2018). HMGCS/HMGCR implications in cancer cell proliferation and cooperation with Ras signaling are currently used in cholesterol-lowering drug therapy (Schwarz et al. 2018). According to Ding et al. (2008), HMGCR is an important marker for tumor testis transformation in mice.

## Conclusion

Mechanisms concerning Leydig cell tumorigenesis are scarcely known, and the role of lipid metabolism in tumor cells has long been disregarded. Very recently, it was suggested that knowing reprogramming of metabolic mechanisms in tumor can be used as prominent future target of therapy (Sreedhar and Zhao 2018). We presented here, for the first time, alterations in lipid- and cholesterol-associated proteins and one possible mechanisms of action of these molecules in LCT, which concomitantly may be primary disturbances in healthy Leydig cell. Further studies are needed to elucidate the type, role, and regulation of lipids synthesized in tumors of steroidogenic cells. Our findings shed light on the novel functional interplay between GPER and PPAR ultimately probably controlling and/or affecting lipid metabolism and steroidogenesis in LCTs. Modifications of expression of LHR, PKA, PLIN, HSL, StAR, TSPO, HMGCS, and HMGCR, together with cGMP and PI3K-Akt-mTOR pathways, may be suitable in developing innovating approaches (combined with transcriptome/proteome analyses and lipidomic data) that target pathological processes of Leydig cells. There is an urgent need for additional experimental and clinical data to complete the current knowledge on the biology and molecular characteristics of LCTs that will guide the early diagnostics, treatment, and surveillance of incoming patients with this disease. Another important issue is elaboration of experimental model that we are currently working on (Gorowska-Wojtowicz et al. 2019) including those more accurately reflecting human tumor, e.g., human tumor Leydig cell lines and human tumor xenografts.
